# A randomised controlled trial of performance review and facilitated feedback to increase implementation of healthy eating and physical activity-promoting policies and practices in centre-based childcare

**DOI:** 10.1186/s13012-019-0865-7

**Published:** 2019-02-18

**Authors:** Meghan Finch, Fiona Stacey, Jannah Jones, Sze Lin Yoong, Alice Grady, Luke Wolfenden

**Affiliations:** 1 Hunter New England Population Health, Wallsend, NSW 2287 Australia; 2 School of Medicine and Public Health, University of Newcastle, Callaghan, NSW Australia; 3 Hunter Medical Research Institute, Newcastle, NSW 2300 Australia; 4 Priority Research Centre for Health Behaviour, University of Newcastle, Callaghan, NSW Australia

**Keywords:** Childcare, Implementation, Nutrition, Physical activity, Obesity prevention, Children

## Abstract

**Background:**

While it is recommended that childcare services implement policies and practices to support obesity prevention, there remains limited evidence to inform policy and practice. The aim of this study is to examine the effectiveness of performance review and facilitated feedback in increasing the implementation of healthy eating and physical activity-promoting policies and practices in childcare services.

**Methods:**

The study was conducted with childcare services in the Hunter New England region of New South Wales, Australia. Eligible services were randomised to a wait-list control group or to receive the implementation strategy. The strategy targeted the implementation of written nutrition, physical activity, and small screen recreation policies; providing information to families regarding healthy eating, physical activity, and small screen time; providing twice weekly healthy eating learning experiences to children; providing water and plain milk only to children; providing fundamental movement skills activities for children every day; and limiting the use of electronic screen time for educational purposes and learning experiences. Intervention services received a performance review and facilitated feedback process five times over the 10 months that included an assessment of current practices, goal setting, identification of barriers to implementation, problem-solving, and resource provision. The primary outcome was the proportion of services implementing all six policies and practices, assessed by nominated supervisor completion of a computer-assisted telephone interview at baseline and 12-month follow-up.

**Results:**

One hundred and eight services took part. There were no significant differences in the proportion of services implementing all six practices at 12 months (mean difference 0.51; 95% CI 0.16 to 1.58; *p* = 0.24). There were also no differences between groups in the mean number of policies and practices implemented (mean difference 0.1; 95% CI − 0.4 to 0.6; *p* = 0.71), or the proportion implementing each of the six individual policies and practices at 12 months (OR range 0.57 to 1.85; *p* > 0.05).

**Conclusions:**

Further support may be required to assist childcare services to make recommended changes to their policies and practices.

**Trial registration:**

The trial was registered retrospectively on 10 September 2014 with the Australian New Zealand Clinical Trials Registry ACTRN12614000972628.

**Electronic supplementary material:**

The online version of this article (10.1186/s13012-019-0865-7) contains supplementary material, which is available to authorized users.

## Background

Overweight and obesity established in childhood has been shown to track into adulthood [1] increasing the future population health burden of the most prevalent causes of adult mortality and morbidity [2, 3]. Inadequate physical activity and poor nutrition are key risk factors related to the development of overweight and obesity [4, 5]. The early childhood years have been recognised as a critical time in the development of dietary intake and physical activity behaviours [6, 7].

The World Health Organization recently identified centre-based childcare services (including organised group pre-school early education programs, infant classes, reception classes, nurseries and long day care) [8] as an important setting for the delivery of population-based interventions in order to prevent childhood obesity [9]. They have the potential to reach a significant proportion of the population aged less than five [10] and existing infrastructure to facilitate, support, and promote child healthy eating and physical activity. In recognition, governments and private organisations both in the United States (US) and Australia have developed specific recommendations regarding the implementation of service policies and practices to improve diet and increase physical activity levels among children attending care [11–14]. Such policies and practices have included a written service nutrition and physical activity policy, the provision of nutrition learning experiences to children, provision of nutrition resources to parents, limiting the provision of sweetened drinks to children, structured physical activity opportunities (e.g. fundamental movement skills programs), and limiting opportunities for sedentary screen time (e.g. computer use).

Despite such recommendations, evidence suggests that healthy eating and physical activity-promoting policies and practices are not routinely implemented by childcare services [13, 15–19]. For example, in the US, studies have reported that half of services do not provide drinking water in classrooms [17], around a quarter serve juice [20], and less than one third have written nutrition policies regarding food consumed by staff [18]. A 2016 study conducted in Canada reported that only 37% of childcare services provide nutrition education/resources to parents (on a regular basis) [21]. Further, in the US, research suggests 47–50% of childcare services do not have a physical activity policy [13, 22] and more than a quarter (28%) do not provide teacher-led physical activity [23], while in Australia, half of services reported not having a physical activity policy (41–48%) and nearly a third (28–30%) allow 3- to 5-year-old children non-active small screen recreation daily [15].

To maximise the public health benefit of obesity prevention interventions delivered in childcare, widespread implementation of recommended policies and practices is required. However, evidence to inform interventions is currently limited [24, 25]. A recently published Cochrane review examined the effectiveness of childcare obesity prevention interventions seeking to improve the implementation of healthy eating and/or physical activity policy and practice change and identified just ten such controlled trials [25]. The review concluded that there was considerable heterogeneity in the implementation strategies tested which precluded isolation of the impact of individual strategies and found weak and inconsistent evidence regarding their effectiveness in improving implementation of obesity prevention policies and practices in this setting [25]. The review identified a need for further implementation research in childcare [25].

Audit and feedback (also known as performance feedback) has long been used to support organisational change, particularly in clinical settings [26]. Performance review and feedback interventions are defined as those where an individual’s professional practice or performance (usually objectively measured health professional practice or patient outcomes) is measured over a specified period of time and compared against professional standards or targets with the results fed back to the individual [27]. A Cochrane review including 140 randomised controlled trials (RCTs), primarily conducted in the outpatient setting to improve prescribing or testing, concluded that audit and feedback interventions resulted in improved professional practice and the quality of health care [27]. The findings indicated that following audit and feedback, desired practice behaviours improved by around 4% on baseline behaviour [27]. The magnitude of the effect of audit and feedback however appears greater when baseline adherence is low, feedback frequency is high, and multiple modes of feedback occur [27]. Additional attractive features of audit and feedback are its capacity to be routinely delivered using modalities, such as telephone that enable reach to large numbers of services at relatively low cost, and ability to address identified implementation barriers such as beliefs about capabilities and motivation [28]. Compared with more resource-intensive interventions delivered under tightly controlled research conditions, the impact of such interventions that are able to be delivered under ‘real world’ conditions are of particular interest to policymakers and practitioners as they are more likely to provide a better approximation of the effect of intervention when delivered in the absence of research support and expertise [28]. Audit and feedback therefore presents as a feasible whole of population approach to supporting childcare services to implement healthy eating and physical activity policies and practices. The primary aim of this study was to examine the effectiveness of a performance review and facilitated feedback intervention in increasing the implementation of healthy eating and physical activity-promoting policies and practices in childcare services.

## Methods

### Design

The study employed a parallel group randomised controlled trial (RCT) design [29]. The trial protocol has been described in detail previously [30]. A sample of eligible childcare services, within the Hunter New England Region of New South Wales (NSW), Australia, was approached to participate. This implementation trial was conducted as part of government-funded health promotion services routinely delivered to all childcare services by a regional population health unit (Hunter New England Population Health (HNEPH)) [31]. The unit is responsible for supporting the implementation of healthy eating and physical activity policies and practices among early childhood education and care services across the Hunter New England government health region [31]. The trial, conducted from August 2013 to December 2014, was registered with the Australian New Zealand Clinical Trials Registry (ACTRN12614000972628) and approved by the Hunter New England Human Research Ethics Committee (06/07/26/4.04) and the University of Newcastle Human Research Ethics Committee (H-2008-0343).

### Participants and recruitment

The study recruited centre-based childcare services in the Hunter New England region. A list of all 366 childcare services in the region provided by the Early Childhood Education and Care Directorate (the Government Licensing Authority) served as the sampling frame. Study information and consent forms were mailed to services approximately 2 weeks before a telephone call from a research assistant assessing eligibility and requesting consent to participate in data collection. Services catering exclusively for children requiring specialist care, mobile preschools, and Department of Education and Communities preschools were excluded as they were outside of the ethics approval, as were services already involved in an alternative RCT currently underway in the Hunter New England region. Services already identified through local health service data as comprehensively implementing healthy eating and physical activity policies and practices were also excluded. Eligible, consenting services were randomly allocated to the intervention or control group.

### Randomisation and blinding

Eligible childcare services were allocated to intervention or control in a 1:1 ratio using computerised random number function undertaken by an independent statistician. Randomisation occurred prior to service consent to participate and baseline data collection. Staff responsible for recruitment of childcare services and data collectors were blinded to group allocation. Childcare services were not made aware of which study group they were allocated to until after baseline data collection.

### Implementation strategy

#### Policies and practices targeted for implementation

We sought to increase service implementation of healthy eating and physical activity policies and practices consistent with best practice Australian healthy eating and physical activity guidelines for the childcare setting [12], and evidence reviews of policies and practices shown to be associated with child healthy eating and physical activity [20, 32]. The policies and practices targeted for implementation included:

1. Service having written nutrition, physical activity, and small screen recreation policies.
2. Service providing information to families (healthy eating, physical activity, small screen time, and breast-feeding, where relevant)
3. Service providing structured and specific learning experiences about healthy eating at least two times per week
4. Service supplying age-appropriate drinks to children (only water and age-appropriate milk)
5. Service conducting fundamental movement skills activities for children aged 3–5 years every day to at least 90% of children
6. Service limiting the use of small screen recreation by children aged 3–5 years to only educational purposes and for learning experiences

The policies and practices were identified and targeted from a broader group of 15 practices for which Hunter New England Population Health was responsible for supporting childcare services to implement as part of a state-wide childhood obesity prevention initiative (Healthy Children Initiative). Policies and practices in this intervention were selected on the basis of existing health service monitoring data summarising policy and practice implementation by childcare services in the region [33]. The sub-groups of targeted practices were included as they were those identified to have low prevalence of implementation.

### Strategies

The implementation intervention was primarily delivered to nominated supervisors of each childcare service via five cycles of performance review and facilitated feedback conducted over a period of 10 months. Formative work including consultations with local childcare services was conducted to identify relevant factors that may impede local implementation of the targeted policies and practices. Damschroder’s Consolidated Framework for Implementation Research (CFIR) was then used to help classify barriers and inform strategy content [34]. Damschroder’s Consolidated Framework for Implementation Research integrates 19 theoretical models and is composed of five major domains identified as influential in successful implementation: innovation characteristics, outer setting, inner setting, characteristics of the individuals involved, and the process of implementation [34]. Table 1 provides a summary of the four targeted constructs and how these were applied.

**Table 1:** Application of the consolidated framework for implementation research

Construct	Application to intervention
Intervention characteristics	
Intervention source: Was the intervention developed internally by childcare services or by external agencies?	The implementation strategies were designed externally by an expert advisory group of health promotion practitioners, psychologists, dietitians, behavioural scientists, and physical activity experts, in consultation with nominated supervisors from local childcare services. Facilitated feedback was provided from a reputable source via experienced government health service support officers known to the services.
Evidence strength and quality: What are the nominated supervisor perceptions of the strength and quality of evidence that the intervention will have the intended outcomes?	Targeted policies and practices were consistent with national mandatory licensing and accreditation requirements and state government evidence-based healthy eating and physical activity best practice guidelines for the setting. These links were communicated to nominated supervisors during telephone contacts and via newsletters.
Adaptability: Is the intervention able to be adapted or tailored to meet the needs of the childcare service?	Telephone discussions included a focus on facilitating adaptation around ways in which practices could be applied in each service (e.g. different methods of communicating with families or integration of structured learning experiences within service routines).
Complexity*:* What are the nominated supervisor perceptions of implementation difficulty?	Telephone discussions focused on integrating policy and practice change within existing service routines to reduce the burden on each childcare service. This was communicated during telephone contact and highlighted via case studies included in newsletters.
Design quality and packaging: What are the nominated supervisor perceptions of how the intervention is presented?	Tools and resources were reviewed by nominated supervisors to ensure that they were visually appealing, professionally presented, and user-friendly during formative work preceding the trial.
Cost: What are the costs of the intervention and associated implementation?	As the implementation strategies formed part of Hunter New England Population Health’s routine service delivery, they were provided at no cost to the service. In addition, suggestions given to services to support policy and practice implementation prioritised low or no cost approaches. These were communicated during telephone contacts and via case studies included in newsletters.
Outer setting	
External policy and incentives: What are the external strategies to spread the intervention (including policy and regulations, external mandates, recommendations, and guidelines)?	Targeted policies and practices were consistent with national mandatory licensing and accreditation requirements and state government evidence-based healthy eating and physical activity best practice guidelines for the setting. The application of continuous quality improvement processes (facilitated reflection, problem-solving, goal setting, and action planning) during telephone contacts also aligns with external accreditation requirements for the child care setting.
Inner setting	
Tension for change: Does the nominated supervisor perceive the current situation as needing to change?	The need for change was demonstrated via individualised feedback reports of policy and practice implementation and advocated by implementation support staff during telephone contacts.
Relative priority: Do childcare service staff have a shared perception of the importance of implementation within the childcare service?	Nominated supervisors nominated supervisors were expected to endorse implementation of the targeted practices and to communicate goals and action plans, as well as progress to service staff.
Organisational incentives and rewards: Does the intervention include incentives such as goal-sharing awards, performance reviews, and increased stature?	Services that demonstrate achievement of all policies and practices received a certificate of recognition and were promoted to other intervention services in newsletters.
Goals and feedback: Are goals clearly communicated, acted upon, and fed back to nominated supervisor?	Facilitated performance feedback was provided to services regarding implementation of targeted policies and practices. Continuous quality improvement processes including review of progress, positive reinforcement, and discussion of deficits identified from feedback reports, problem-solving, goal setting, and action planning were incorporated into the telephone contacts.
Leadership engagement: Are nominated supervisors committed, involved, and accountable for the implementation?	Nominated supervisors were encouraged to circulate feedback reports to management committees and childcare service staff.
Access to information and knowledge: How easy is it for nominated supervisors to access information and knowledge about the intervention and how to incorporate it into work tasks?	Nominated supervisors received resources and ongoing support from implementation support staff via scheduled telephone contacts and email. Services were also provided with contact details for implementation support staff and encouraged to follow-up at any time for advice or assistance and all resources were made available via the program website.
Process	
Engaging: Are appropriate individuals involved in the implementation through education, role modelling, and training?	Nominated supervisors were directly engaged in implementation through telephone discussions regarding service priorities, service goals, and strategies to meet goals and overcome barriers. Nominated supervisors were also encouraged to communicate and endorse practice changes to service educators.
External change agents: Are individuals available who are affiliated with an outside entity who facilitate intervention decisions in a desirable direction?	Facilitated feedback and implementation support was provided from a reputable source via experienced government health service support officers known to the services.

The initial performance review was completed in-person by trained support officers (1.5-h duration). Detail regarding the content of the visit is described in the protocol paper [30]. The four subsequent reviews were completed via telephone (30-min duration). Support officers had qualifications in nutrition, exercise physiology, psychology, and health education, with at least 2 years’ experience working with childcare centres. Each performance review included a standard assessment of the implementation of the targeted policy and practice (completed via telephone by support officers). Facilitated feedback included a discussion of current implementation status, assessed on the basis of information reported by the nominated supervisor using standard criteria with support tailored to meet service needs based on the policies and practices not being implemented. During each contact, implementation support staff facilitated a discussion regarding how to implement policies and practices to best suit the service needs and advice provided on the use of tools and resources was tailored based on identified barriers in line with service identified priorities. Where services were already meeting a policy or practice, implementation support was directed towards policies and practices not yet achieved. A follow-up email was then sent after the contact summarising agreed actions and providing resources.

Based on reviews of empirical research evidence, we aimed to incorporate components shown to be associated with greater effect in audit and feedback interventions targeting professional practice change. A detailed outline of the evidence and description of how it was applied to the intervention in practice is reported in the protocol paper [30]. In brief, these included providing feedback from a reputable source via experienced government health service support officers known to the services [26]; multimodal feedback provided in person (at the initial visit), via telephone, and in written form [26]; feedback tailored to each individual service context and identified implementation barriers; and use of reinforcement, facilitated reflection, problem-solving, goal setting, and action planning [26, 35–38]. Support officers monitored and recorded intervention delivery in a database. In addition, nominated supervisors were expected by service staff to offer support for the implementation of the targeted practices and to communicate goals and action plans, as well as progress.

Intervention services were provided with resources to support the implementation of these policies (policy templates, DVD, manuals, posters, and parent lunchbox resources). The policy templates were word documents to enable the service to amend to suit the needs of their service and provided an example aim, rationale with sample strategies that supported the recommended practices. They also referred to relevant childcare standards and regulations and provided a prompt for a timeframe for policy review. The DVD described fundamental movement skills and demonstrated children practicing and educators engaging in intentional teaching of these skills. Posters encouraged healthy eating and physical activity in young children, for example, healthy drinks and active play (available from http://www.health.gov.au/internet/main/publishing.nsf/Content/phd-gug-posters). Lunchbox resources included a parent brochure providing a list of recommended foods to be packed including recipe ideas and label reading. Program resources are available from http://www.goodforkids.nsw.gov.au/early-childhood-services/. Services were also emailed four electronic newsletters which communicated key messages relating to the targeted healthy eating and physical activity policies and practices [39], provided web links for additional information, and included case studies from individual services reporting successful approaches and strategies to improve or sustain policy or practice implementation or describing new or novel problem-solving approaches to alleviate service implementation barriers.

### Comparison

Control group services received the same four electronic newsletters (described above) during the intervention period, but did not receive any other resources. Staff could implement any of the recommended practices described in the newsletter, but did not receive support in doing so. At completion of the intervention period, control services were offered the complete intervention.

### Outcomes

Computer-assisted telephone interviews (CATI) were used to collect data at baseline and 12-month follow-up. The interviews were conducted as part of government-funded service delivery with baseline and follow-up data collection aligned with 12-monthly routine practice assessment. The survey was undertaken with the nominated supervisor or lead educator at each service. In Australia, nominated supervisors are responsible for policy development, ensuring compliance with licensing and accreditation requirements and most also have teaching roles. The surveys assessed service characteristics and implementation of healthy eating and physical activity policies and practices [40] and took approximately 15–25 min to complete. CATI interviewers were blind to study group allocation (Additional file 1).

The primary outcome was the change in prevalence of services implementing all six targeted healthy eating and physical activity policies and practices at 12 months. Twelve items assessed the implementation of six policies and practices. The items have been validated and used in previous trials [15, 40–43] and included:

1. Services having written nutrition, physical activity, and small screen recreation policies (three items) (all three) (which may be combined with another policy) (yes/no to having all three).
2. Service providing information to families on healthy eating, physical activity, small screen time, and breast-feeding, where relevant (four items) (all topics). Information was required to be distributed at least once in the last 12 months and could include material handed directly to parents, mailed or emailed or placed in their child’s pigeon hole or bag, or information included in newsletters or at orientation (yes/no to all topics). Examples could include a list of recommended foods for lunchboxes and lunchbox ideas, physical activity and screen time recommendations for children, and breastfeeding guidelines.
3. Service providing structured and specific learning experiences about healthy eating at least two times per week (one item, yes/no). Examples included experiential activities about food, cooking skills, food growing (e.g. kitchen/vegetable gardens, planting seeds), tasting sessions, and discussion around “everyday” and “sometimes” foods.
4. Service only supplying age-appropriate drinks to children including water and reduced fat plain milk (one item) (yes/no). For children less than 2 years old, the service reported supplying plain full-fat milk.
5. Service conducting fundamental movement skills activities for children aged 3–5 years every day to at least 90% of children (two items). Where service reports the average number of days per week that educators lead structured activities to develop Fundamental Movement Skills (could be during transition activity, group or circle time, or during outdoor play) is equal to all service opening days, and where service estimate of the percent of children that usually participate is 90% or greater (or are encouraged to participate if special needs).
6. Service limiting the use of small screen recreation (one item) (i.e. TVs, videos, DVD, computers and other electronic games, iPads/tablets) by children aged 3–5 years to only educational purposes and for learning experiences such as to gain knowledge or share information about a specific learning area or child’s interest or to facilitate exploration of activity, dance, or movement (yes/no).

To provide greater description of changes occurring in the measures of practice implementation, we also report the proportion of services that implemented each of the policies and practices at baseline and 12 months, the mean number of practice services were compliant with, and the breakdown for each individual policy and for information home to families. These measures were not prospectively registered.

Service characteristics included service type, days open, ages catered for, hours of operation, number of children enrolled, and number of primary contact teaching staff. Intervention participants also completed a survey about their satisfaction with the intervention components including program resources, feedback reports, support calls, and newsletters at completion of the intervention. Eleven items were assessed using a 7-point Likert scale (1 = strongly disagree to 7 = strongly agree). Data regarding intervention fidelity was sourced from program records which were collected by research staff during implementation.

### Sample size calculation

A sample size of 152 services (76 per group) can detect a difference of 20% in the prevalence of services implementing all targeted policies or practices with 80% power and alpha of 0.05, assuming 10% of control services implement all targeted policies and practices at follow-up. A difference of 20% was deemed by the Population Health Unit (HNEPH) responsible for improving policy and practice implementation as meaningful from a public health service delivery perspective and similar to policy and practice changes observed in previous studies conducted by the research team in this setting [42, 43].

### Statistical analysis

All statistical analysis was performed using SAS v9.2 statistical software. Descriptive statistics were used to describe the demographic and service characteristics of the study sample. Sample characteristics were compared using Fisher’s exact test (percentages) and the *t* test (means). Intervention effectiveness was determined using logistic regression, controlling for baseline. The primary analysis was based on an intention-to-treat framework. Sensitivity analysis was undertaken using imputation of baseline values for missing follow-up data [44]. Additional sub-group analyses of the primary trial outcome were pre-specified and undertaken by childcare service geographic location (urban [regional cities and inner regional areas] or rural [outer regional and remote areas]) as determined by the Australian Standard Geographic Classification. Socioeconomic status was classified according to the Socioeconomic Indices for Areas, and services were grouped by postcode as being in the top 50% of New South Wales or the bottom 50% of New South Wales. All statistical tests were two-tailed with an *α* of 0.05.

## Results

### Response rates and sample characteristics

**Fig. 1:**
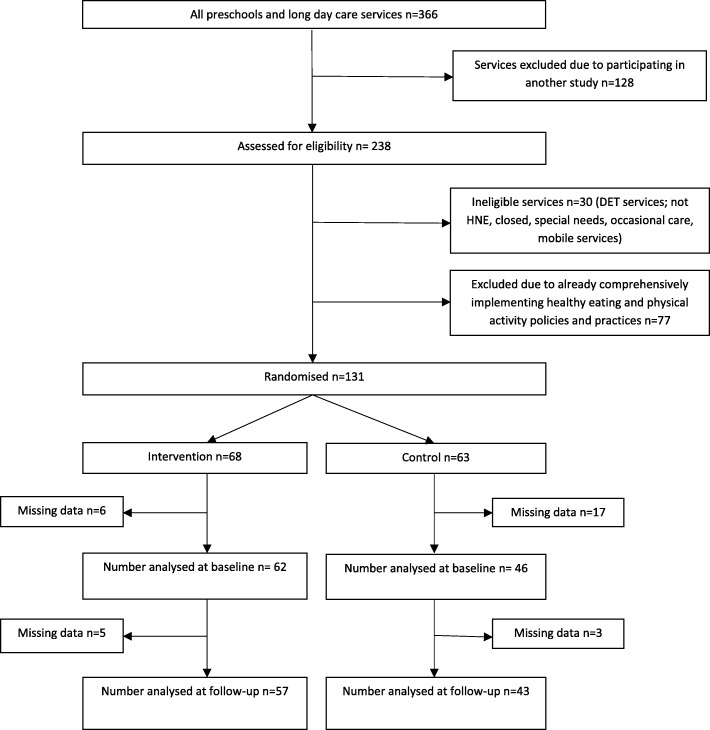
Participant flow chart

Figure 1 describes the participation of services in the trial. Of the 366 childcare services in the region, 128 were excluded given their involvement in an alternative RCT, a further 30 did not meet inclusion criteria, and an additional 77 were identified as comprehensively implementing healthy eating and physical activity policies and practices. A total of 131 services were randomised among which 68 were allocated to intervention and 63 to the control. Six services allocated to the intervention and 17 services allocated to the control group did not provide baseline data and were therefore excluded. There were 62 intervention services and 46 control services that provided baseline data, giving a consent rate of 82%. At 12-month follow-up, five intervention participants and three control participants were unavailable to complete follow-up.

### Service characteristics

Service characteristics by intervention and control group are shown in Table 2. Intervention groups were similar at baseline. The majority of services were long day care services (73.0%), and one third were preschool services (32.8%). Almost all services were open for 5 days per week (93.0%). Food was provided by the service for the children in 60% of services and by the family in 34% of services. Services were open for an average of 9 h per day. The majority of services in both groups were located in a metropolitan or inner regional city (61.4%) with 38.7% classified as outer regional or remote areas.

**Table 2:** Baseline demographic comparison of study groups

Characteristic	Control *n* = 46		Intervention *n* = 62	
	*N*	%	*N*	%
Type of service^a^				
Long day care service	33	71.7	46	74.2
Pre-school service	16	34.8	19	30.7
Provides food for meals and snacks				
Families provide all food	18	39.1	18	29.0
Both family and service provides food	2	4.4	6	9.7
Service provides all food	26	56.5	38	61.3
Number of days open per week				
< 5 days	5	10.9	2	3.2
5 days	41	89.1	60	96.8
Service caters for:				
Children under 1 year	32	69.6	44	71.0
1 year olds	32	69.6	43	69.4
2 year olds	35	76.1	46	74.2
3–5 year olds	46	100	62	100
Number of hours of operation, mean (SD)	9.5 (2.0)		9.97 (2.1)	
Number of children enrolled, mean (SD)	116 (142)		105 (54)	
Number of (primary contact) teaching staff, mean (SD)	10.6 (5.3)		11.9 (6.4)	
Service geographic location				
Urban	27	61.4	38	61.3
Rural	17	38.6	24	38.7
Service socio-economic area				
Top 50% of New South Wales	16	36.4	30	48.4
Lower 50% of New South Wales	28	63.6	32	51.6

^a^Three control services and three intervention services identified as both a preschool and long day care service; therefore, percentages add to more than 100%

### Implementation of healthy eating and physical activity policies and practices

#### Primary trial outcome

There were no services that were implementing all of the six policies and practices at baseline, and few who were meeting all six practices at follow-up (9.7% [*n* = 6] intervention services and 17.4% [*n* = 8] control services) (mean difference 0.51; 95% CI 0.16 to 1.58; *p* = 0.24) (Table 3). Sensitivity analyses carrying baseline observation forward for missing data were also non-significant. There were no significant differences between groups on the primary trial outcome at follow-up by socioeconomic (mean difference 1.25; 95% CI 0.12 to 12.84; *p* = 0.85) or geographic subgroups location (mean difference 0.55; 95% CI 0.05 to 5.85; *p* = 0.62).

**Table 3:** Proportion of services implementing policies and practices at baseline and follow-up

Healthy eating and physical activity-promoting policies and practices	Intervention *n* (%)		Control *n* (%)		Intervention vs control (adjusted for baseline)	
	Baseline (*N* = 62)	Follow-up (*N* = 62)	Baseline (*N* = 46)	Follow-up (*N* = 46)	Odds ratio (95% CI)	*X*^2^ p
Service implementing all six policies/practices	0	6 (9.7%)	0	8 (17.4%)	0.51 (0.16 to 1.58)	0.24
Service having written nutrition, physical activity, and small screen recreation policies	26 (41.9%)	37 (59.7%)	14 (30.4%)	24 (52.2%)	1.18 (0.52 to 2.67)	0.69
Written nutrition policy	62 (100%)	62 (100%)	45 (97.8%)	45 (97.8%)	–^a^	0.93
Written physical activity policy	44 (71.0%)	49 (79.0%)	26 (56.5%)	34 (73.9%)	1.02 (0.38 to 2.72)	0.97
Written small screen recreation policy	32 (51.6%)	43 (69.4%)	19 (41.3%)	27 (58.7%)	1.41 (0.59 to 3.40)	0.44
Service providing information to families on healthy eating, physical activity, small screen time, and breast-feeding, where relevant	14 (22.6%)	31 (50.0%)	11 (23.9%)	17 (37.0%)	1.85 (0.81 to 4.22)	0.14
Healthy eating information	57 (91.9%)	62 (100%)	44 (95.7%)	44 (95.7%)	–^a^	0.66
Physical activity information	41 (66.1%)	55 (88.7%)	28 (60.9%)	33 (71.7%)	3.14 (1.09 to 9.06)	0.03
Small screen recreation information	24 (38.7%)	47 (75.8%)	21 (45.7%)	28 (60.9%)	2.17 (0.93 to 5.07)	0.07
Breastfeeding information	27 (61.4%)	24 (54.6%)	16 (50.0%)	12 (37.5%)	1.86 (0.71 to 4.83)	0.20
Service providing structured and specific learning experiences about healthy eating at least two times per week	52 (83.9%)	50 (80.7%)	39 (84.8%)	39 (84.8%)	0.75 (0.26 to 2.15)	0.59
Service supplying only age-appropriate drinks to children (only water and age-appropriate milk)	37 (59.7%)	43 (69.4%)	30 (65.2%)	31 (67.4%)	1.22 (0.51 to 2.91)	0.66
Service conducting fundamental movement skills activities for children aged 3–5 years every day to at least 90% of children	32 (51.6%)	35 (56.5%)	25 (54.4%)	32 (69.6%)	0.57 (0.25 to 1.28)	0.17
Service limiting use of small screen recreation by children aged 3–5 years to only educational purposes and for learning experiences.	49 (79.0%)	54 (87.1%)	36 (78.3%)	38 (82.6%)	1.42 (0.48 to 4.19)	0.52

^a^Due to the high proportion who answered yes, it is not possible for the odds ratios to be modelled

Table 2 shows the proportion of services that were compliant with each policy or practice at baseline and 12-month follow-up. At baseline, the proportion of services already implementing healthy eating learning experiences and limiting the use of small screen recreation was high. Between baseline and follow-up, there were increases in compliance for all policies and practices in both intervention and control services, with no significant differences between groups. The largest increases over time were observed for intervention services providing information to families (by 27.4%) and the service having written policies (by 17.8%).

#### Other outcomes

There were no differences between the groups in the number of practice services were compliant with (mean difference 0.1; 95% CI − 0.4 to 0.6; *p* = 0.71). At baseline, intervention services were compliant with a mean of 3.4 (95% CI 3.1 to 3.6) practices, which increased to 4.0 practices at follow-up (95% CI 3.8 to 4.3). Control services were compliant with 3.4 practices at baseline (95% CI 3.1 to 3.6) and increased to 3.9 practices at follow-up (95% CI 3.5 to 4.4).

### Intervention fidelity and acceptability

Overall, more than half (56.5%; *n* = 35) of services participated in all five performance review and feedback cycles (contacts). Participation rate for each of the five contacts ranged between 83 and 93% (*n* = 52–58). The mean number of contacts was 3.56 (SD 1.58) and range 0 to 5. Most services reported that intervention components were helpful (Table 4) and that they were happy with the frequency of support calls and e-newsletters.

**Table 4:** Acceptability of the intervention

Domain	Measure	Mean	SD
Program resources	Resources were helpful to implement program (mean rating)	6.6	0.63
	Resources were relevant (mean rating)	6.7	0.59
Feedback reports	Reports assisted with communicating progress about the program to service staff (mean rating)	6.3	1.10
	Program reports helped engage staff in implementing the program (mean rating)	6.3	1.10
Support calls	Support calls were helpful (mean rating)	6.3	1.14
	Support calls were motivating (mean rating)	6.3	1.12
	Happy with the frequency of support calls (%)	83%	
	Would have preferred to receive the support calls less frequently (%)	15%	
Newsletters	E-newsletters were useful (mean rating)	6.1	1.08
	Happy with frequency of e-newsletters (%)	75%	
	Would have preferred to receive the newsletters more frequently (%)	23%	

Rating system was 1 = strongly disagree to 7 = strongly agree

## Discussion

This study sought to test an audit and facilitated feedback intervention to improve the implementation of healthy eating and physical activity-promoting policies and practices in the childcare setting. The study found the intervention was not effective in improving, relative to control, the implementation of targeted policies and practices. Findings indicate that such an intervention in isolation may not be sufficient to improve childcare environments, suggesting policymakers and practitioners may require more intensive strategies to support practice change in this setting.

Study findings contrast with previous research in the field [25]. Three targeting the implementation of healthy eating and physical activity practices included in a recent Cochrane review trials tested multi-component implementation strategies (for example, staff workshops, policy support, resources, and follow-up contact/support) capable of addressing a number of barriers to change [45–47] and reported significant improvements in measure of policy or practice implementation. The lack of evidence of improvement in implementation found in the current study suggests future strategies may need to address a more comprehensive range of barriers to that achieved through audit and facilitated feedback (e.g. beliefs about capabilities and consequences) alone [48]. For example, views of parents and childcare service management are important in the implementation of obesity prevention practices, as is the accessibility of external resources to support implementation [49].

The potential impact of the implementation strategy may have been hindered by targeting nominated supervisors of childcare services. While nominated supervisors are ultimately responsible for service operations, actual implementation of obesity prevention practices requires the action of service staff. In this trial, we did not assess the extent to which nominated supervisors engaged with childcare service staff to support service improvement as a result of the intervention. Failure of the nominated supervisor to do so may have reduced the potential impact of the implementation strategy as previous research suggests that implementation is more likely to occur if appropriate individuals or groups are engaged in the change process [34]. The use of strategies to ensure greater penetration of performance review and feedback across childcare service staff may therefore be required [34]. Targeting multiple policies and practices may have also reduced the potential effect of the implementation strategy in this trial compared to targeting a single policy or practice. Interventions perceived as complex and placing greater demand on childcare services and their staff are less likely to be implemented [34]. In settings such as schools, strategies targeting single health policies have yielded large improvements in implementation [49]. Such an approach could be considered for future implementation initiatives in childcare.

Furthermore, the introduction of the National Quality Standards for the setting (including nutrition and physical activity elements) in the 12 months prior to the commencement of the trial and broad government support for implementation of policies and practices occurring state-wide may have also facilitated policy and practice implementation in the control group potentially reducing the likelihood of an intervention effect.

The trial used random assignment and blinding of outcome assessor to improve internal validity and used probability sampling, less stringent eligibility criteria, and was conducted in the context of a service delivery initiative, maximising external validity. However, there were a number of limitations. First was the use of phone survey for audits conducted remotely (off-site) and reliant on nominated supervisor self-report of implementation outcomes. While validated and used in previous trials, such measures are prone to reporting bias such as social desirability bias [50]. The use of on-site observation or measurement triangulation techniques should be considered for future intervention to improve the robustness of audit and research findings [51]. Secondly, the quantitative nature of the outcome measure limited our capacity to collect contextual information that may have provided greater insight into any systematic or structural barriers to implementation. Third, outcomes were assessed post-intervention at one time point only. Additional time points would allow for more sophisticated analyses and potential assessment of sustainability. Furthermore, there is possible bias in the sample given that potential participants had already declined to participate in another intervention. This may indicate the participating services may have been less likely to adopt the policies and practices targeted in the intervention. In addition, the study was under-powered. Finally, the measure used to assess the practices may not have been sensitive enough to detect changes in implementation.

Notwithstanding study limitations, the trial makes an important contribution to a limited evidence base on the effectiveness of performance review and feedback as a standalone implementation strategy. While self-assessment, audit, or feedback is a common element of most previously trialled strategies to improve implementation of nutrition and physical activity policies and practices [25], in isolation audit and facilitated feedback does not appear to be sufficient to achieve implementation improvements. For policymakers and practitioners, the findings underscore the need for consideration of additional implementation strategies in future trials to maximise obesity prevention initiatives in this setting. Strategies that address the reported barriers to implementation that include specific behaviour change techniques [48] (e.g. prompts/cues, material reward, habit formation) or accounting for policy context (e.g. changing licensure requirements) are two areas that may offer promise.

## Conclusion

A performance review and facilitated feedback intervention was not effective in improving the implementation of healthy eating and physical activity-promoting policies and practices within childcare services. Future research targeting multiple health policies or practices in this setting should include additional implementation support. Gaps remain in available evidence to inform programs delivered in real-world conditions aiming to support the implementation of healthy eating and physical activity policies and practices as part of routine practice in childcare services [52].

## Additional file

Additional file 1: Computer Assisted Telephone Survey. (DOCX 27 kb)

## Abbreviations

CATI: Computer-assisted telephone interview
CFIR: Consolidated Framework for Implementation Research
HNEPH: Hunter New England Population Health
RCT: Randomised controlled trial
US: United States
