# Supplementary material for: A randomised controlled trial of performance review and facilitated feedback to increase implementation of healthy eating and physical activity-promoting policies and practices in centre-based childcare
Source: Implement Sci. 2019 Feb 18;14:17. doi: 10.1186/s13012-019-0865-7 (PMC6380016; doi:10.1186/s13012-019-0865-7)
Supplement: Supplementary file 1 — Computer Assisted Telephone Survey. (DOCX 27 kb) [file 13012_2019_865_MOESM1_ESM.docx]

**APPENDIX A**

HNE Kids Healthy Eating and Physical Activity Program

HCI Intervention Follow up CATI

September 2014

**Telephone survey items**

| Confirm name of Nominated Supervisor |
| --- |
| Position of person responding to interview |
| Confirm postal address, fax and email |
| Is your service part of a DEC primary or central school? |
| Does your service solely cater for children with special needs? |
| Overall, how many allocated places for children do you have at your service? |
| Overall, how many children are enrolled at your service? |
| Are you aware of any children of Aboriginal or Torres Strait Islander origin enrolled at your service? |
| How many children of Aboriginal or Torres Strait Islander origin are enrolled at your service? |
| Which of the following best describes your service?  1 Preschool  2 Long day care centre  3 Occasional Care |
| Can I confirm that you are still open (^opendays^) days a week? |
| What are your service hours of operation? |
| Which of the following age groups does your service care for?  (Please select all that apply)  1 Children under 1 year  2 1 year olds  3 2 year olds  4 3 to 5 year olds |

Healthy eating and physical activity practices

| Question # in previous CATI |  |
| --- | --- |
| Q6 | Do families provide food for any meals or snacks when their child  attends your service?  [INTERVIEWER NOTE: If yes, prompt: is that just main meals, just snacks,  or all food]  1 Yes, all meals and snacks  2 Yes, some meals and snacks  3 No, service provides all meals & snacks  4 Don't know [DO NOT READ OUT]  .R Prefer not to say [DO NOT READ OUT] |
| Lunch box services  Q10  Q10a | Does your service monitor lunchboxes?  How often do educators monitor the lunchboxes of all children to check the food or drinks packed by families? |
| Guideline  (if yes)  provide | Does your service have written nutritional guidelines for families regarding recommended food and drinks brought from home for meals and snacks?  In the last 12 months, has your service provided all families with a copy of the nutritional guidelines regarding recommended foods and drinks brought from home? |
| Menu  Services  Only.  menu  t_day | The next questions are about the food and drinks on your service's menu.  It would be great if you could please refer to last week's menu when you answer these questions.  Do you have a copy of LAST WEEK'S menu to refer to?  On a typical day, what meals and snacks would your service provide to  children?  [INTERVIEWER NOTE: Read out all response options]  1 Breakfast  2 morning tea  3 lunch  4 afternoon tea  5 dinner  6 Other (Please specify on next screen) |
| Menu services only  Q7  Q8 | In the past week, how often did you serve fruit, including fresh frozen or canned fruit in natural juice, BUT NOT INCLUDING juice or fruit drinks?  1 Never  2 Rarely  3 2 times per week or less  4 3-4 times per week  5 1 time per day  6 2 or more times per day  7 Don't know [DO NOT READ OUT]  8 Prefer not to say [DO NOT READ OUT]  In the past week, how often did you serve vegetables, including fresh frozen or canned, BUT NOT including chips, french fries or potatoes that your service cooks in oil?  1 Never  2 Rarely  3 2 times per week or less  4 3-4 times per week  5 1 time per day  6 2 or more times per day  7 Don't know [DO NOT READ OUT]  8 Prefer not to say [DO NOT READ OUT] |
| Q9 -  Menu Services only | In the last week which of the following foods, if any, did your service provide during the day? This includes for snacks or at meals.  Please select all that apply  1 Fruit or vegetable pieces or platters  2 Confectionary, chocolate, ice cream  3 Fruit bread (e.g. raisin toast), English or  [CONT] fruit muffins or pikelets  4 Iced or creamed cakes, lamingtons or donuts  5 Wholegrain or rice crackers or rice cakes  6 Potato chips, corn chips, cheese flavoured snacks  [CONT] (such as Twisties or Cheezels).  7 Plain popcorn (no added fat),  [CONT] oven baked chips (not oiled)  8 Unsalted pretzels  9 Salted pretzels  10 French fries, hash browns, hot chips (cooked in oil)  11 Dairy snacks (such as yoghurt, cheese, custard)  12 Sweet biscuits with chocolate or cream filling  13 None of the above  14 Don't know [DO NOT READ OUT]  15 Prefer not to say [DO NOT READ OUT] |
| Q11, Q12  (all services) | What drinks, if any, does your service provide during the day? Please select all that apply.  [INTERVIEWER NOTE: Read out all response options]  1 Fruit juice or fruit drink including 100% fruit juice  2 Cordial  3 Water  4 Plain milk  5 Flavoured milk  6 Soft drink  -7 No drinks provided  -8 Don't know [DO NOT READ OUT]  -9 Prefer not to say [DO NOT READ OUT]  What type of plain milk do you provide for children 2 years of age and older? Please select all that apply.  1 Full cream  2 Reduced Fat (including lite, low fat and no fat milk)  -3 Don't know [DO NOT READ OUT]  -4 Prefer not to say [DO NOT READ OUT] |
| Q13 | How often are structured and specific learning experiences about healthy eating implemented as part of your curriculum/program (e.g. vegetable gardens, cooking or tasting sessions, stories or songs about food)  [Interviewer note: this also includes experiential activities about food,  such as food growing, planting seeds, discussion around 'everyday' and  'sometimes' foods, and puzzles and books about food]  1 Never  2 Rarely  3 Monthly  4 Once per week  5 2-4 times per week  6 Daily (or every day the service is open)  7 Don't know [DO NOT READ OUT]  8 Prefer not to say [DO NOT READ OUT] |
| Q15, Q16 | On average how much time each day, do children spend participating in educator-led structured active play, such as circle time, music, dancing or planned activities to develop movement skills?  On average, how much time each day do children have available to spend in child-initiated, free physically active play? This includes both indoor and outdoor free active play. |
| Q17, Q17a | On how many days in the last week did your service Educators lead structured activities to develop Fundamental Movement Skills for all children at your service?  This could have been during a transition activity, group or circle time, or during outdoor play.  1 Never  2 1 Day  3 2 Days  4 3 Days  5 4 Days  6 Everyday (or every day the service is open)  7 Don't know [DO NOT READ OUT]  8 Prefer not to say [DO NOT READ OUT]  On days where structured activities to develop Fundamental Movement Skills occurred, what percentage of the 3 to 5 year olds at your service would usually participate? |
| SSR2 | On average, on how many days each week would children spend time watching television, videos or DVDs or ALSO using computers and other electronic games, iPads or tablets?  1 Never  2 Less than 1 day a week  3 1 day  4 2 days  5 3 days  6 4 days  7 Everyday (or every day the service is open)  8 Don't know [DO NOT READ OUT]  .R Prefer not to say [DO NOT READ OUT] |
| SSR2a,  SSR2b | For which of the following purposes do children aged 3 to 5 years at your  service spend time watching television, videos or DVDs, or using computers,  electronic games or iPads/tablets? Please select all that apply.  [INTERVIEWER NOTE: Read out all response options]  - read 'info re:' as 'INFORMATION ABOUT A specific learning area'  1 To gain knowledge/share info re:a specific learning area  2 For child amusement, enjoyment or entertainment  3 To facilitate exploration of activity, dance or movement  4 For "down time" or "quiet time"  5 For another purpose  -6 Don't know [DO NOT READ OUT]  -7 Prefer not to say [DO NOT READ OUT]  Please specify the other purpose SSR use - other purpose |
| Q14 | How many days in the past week were babies (birth - 12 months of age)  provided with supervised floor-based play time where they were on  their tummies?  1 Never  2 1 Day  3 2 Days  4 3 Days  5 4 Days  6 Everyday (or every day the service is open)  7 Don't know [DO NOT READ OUT]  8 Prefer not to say [DO NOT READ OUT] |
| Q20 | Does your service have a written nutrition policy?  [Interviewer note: this can be combined with another policy]  1 Yes  2 No  3 Don't know [DO NOT READ OUT]  4 Prefer not to say [DO NOT READ OUT] |
| Q21 | Does your service have a written physical activity policy?  [Interviewer note: this can be combined with another policy]  1 Yes  2 No  3 Don't know [DO NOT READ OUT]  4 Prefer not to say [DO NOT READ OUT] |
| Q22 | Does your service have a written policy restricting child viewing of TV, DVDs or Videos?  [Interviewer note: this can be combined with another policy]  1 Yes  2 No  3 Don't know [DO NOT READ OUT]  4 Prefer not to say [DO NOT READ OUT] |
| Q25 Q25a | In the last 12 months, have you sent information home to families from a recognised health authority about any of the following topics?  This would include material handed directly to parents, mailed or emailed or placed in their child's pigeon hole or bag, or information included in newsletters or at orientation. Please select all that apply.  [INTERVIEWER NOTE: Read out all response options]  1 Immunisation  2 HE for children (inc list of foods for lunchboxes/lunch ideas)  3 Physical activity for children  4 Oral hygiene for children  5 Limiting screen time for children  -6 No information is provided  -7 Don't know [DO NOT READ OUT]  -8 Prefer not to say [DO NOT READ OUT]  In the last 12 months, have you sent information home to families from a recognised health authority about breastfeeding?  1 Yes  2 No  3 Don't know  .R Refused |
| educ & Q26a | Do you still have ^educator^ primary contact educators at your service?  If no: How many primary contact educators are working at your service? |
| Q27 | How many of your primary contact educators have received training since 2009 regarding promoting child healthy eating?  This includes training provided by an external agency, or by other trained staff in your service. |
| Q28 | How many of your primary contact educators have received training since 2009 regarding promoting child physical activity?  This includes training provided by an external agency or by other trained staff in your service. |
| Q24 | Each year, does your service monitor and report, internally or externally,  on its achievement of the healthy eating and physical activity objectives  as stated in written policies, guidelines, or other documents?  [Interviewer note: eg. Annual reports ; Business / Service plans; Quality improvement Plan/s; Munch & Move action plan; Service daily diaries  (for families to view), or Reflection of achievements in a Service newsletter]  1 Yes  2 No  3 Service doesn't have HE/PA objectives  4 Don't know [DO NOT READ OUT]  5 Prefer not to say [DO NOT READ OUT] |
